# Supplementary material for: Transient hepatic reconstitution of trophic factors enhances aged immunity
Source: Nature. 2025 Dec 17;650(8101):481–9. doi: 10.1038/s41586-025-09873-4 (PMC12893904; doi:10.1038/s41586-025-09873-4)
Supplement: Supplementary file 1 — This file contains Supplementary Figs. 1–6. [file 41586_2025_9873_MOESM1_ESM.pdf]

nature portfolio

https://doi.org/10.1038/s41586-025-09873-4

Supplementary information

Transient hepatic reconstitution of trophic factors enhances aged immunity

In the format provided by the authors and unedited

Transient hepatic reconstitution of trophic factors enhances aged immunity

Mirco J. Friedrich[1,2,3,4,5], Julie Pham[1,2,3,4,5], Jiakun Tian[2,6], Hongyu Chen[2,6], Jiahao Huang[2,6], Niklas Kehl[2], Sophia Liu[2,7], Blake Lash[1,2,3,4,5], Fei Chen[2,8,9], Xiao Wang[2,6,10], Rhiannon K. Macrae[1,2,3,4,5], and Feng Zhang[1,2,3,4,5*]

Affiliations: (1) Howard Hughes Medical Institute, Cambridge, MA 02139, USA; (2) Broad Institute of MIT and Harvard, Cambridge, MA 02142, USA; (3) McGovern Institute for Brain Research at MIT, Cambridge, MA 02139, USA; (4) Department of Brain and Cognitive Science, Massachusetts Institute of Technology, Cambridge, MA 02139, USA; (5) Department of Biological Engineering, Massachusetts Institute of Technology, Cambridge, MA 02139, USA; (6) Department of Chemistry, Massachusetts Institute of Technology, Cambridge, MA, USA; (7) Ragon Institute of MGH, MIT and Harvard, Cambridge, MA 02139, USA; (8) Harvard Stem Cell Institute, Cambridge, MA, USA; (9) Department of Stem Cell and Regenerative Biology, Harvard University, Cambridge, MA 02138, USA; (10) Stanley Center for Psychiatric Research, Broad Institute of MIT and Harvard, Cambridge, MA, USA.

* Correspondence should be addressed to F.Z. (zhang@broadinstitute.org).

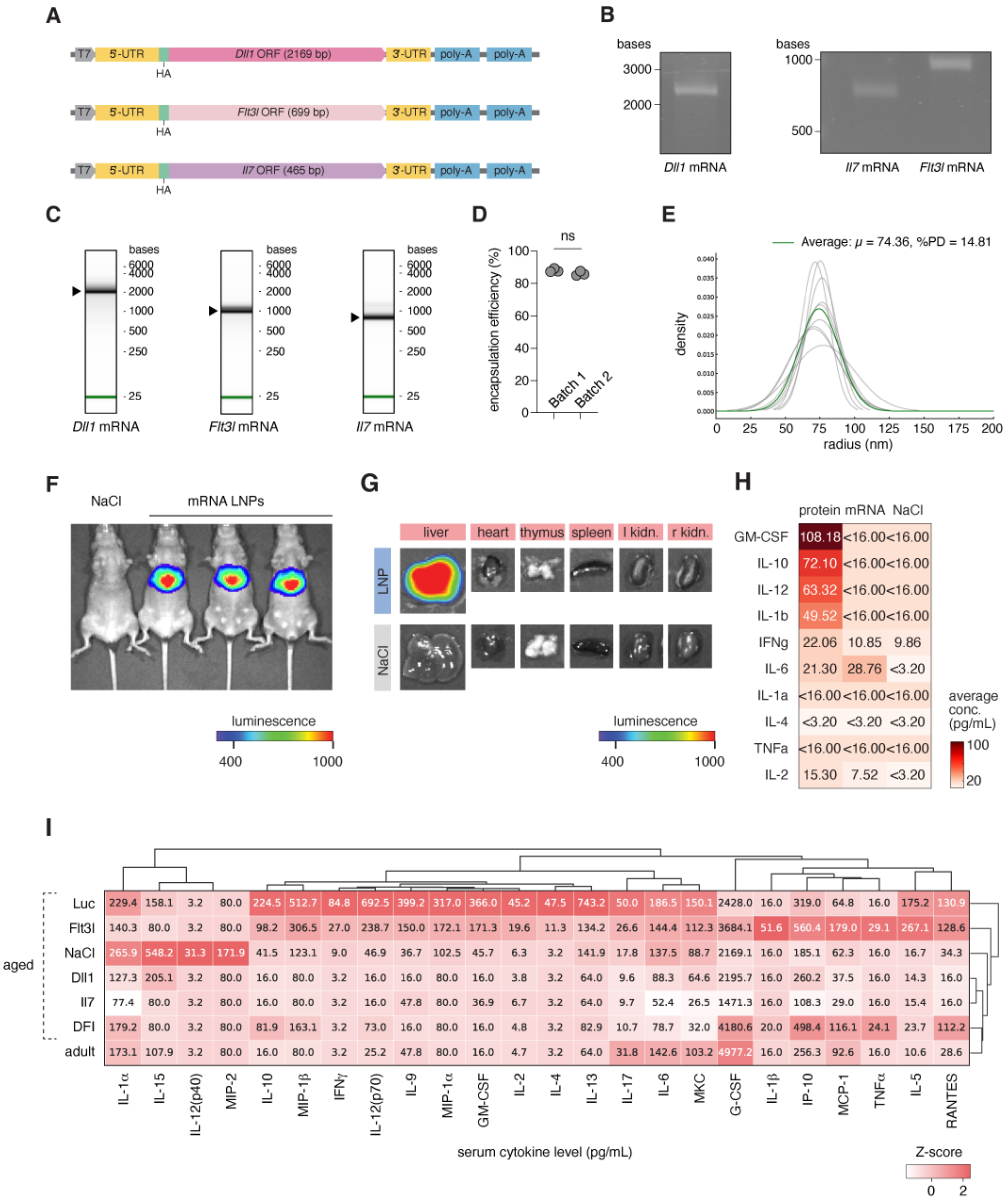

Supplementary Fig. 1 | Formulation, characterization, and biodistribution of DFI mRNA-lipid nanoparticles (LNPs).

(A) Maps of linearized plasmids used for *in vitro* transcription (IVT) of DFI mRNAs.

(B) Gel electrophoresis of *in vitro*-transcribed and capped DFI mRNAs.

(C) TapeStation RNA ScreenTape analysis of *in vitro*-transcribed and capped DFI mRNAs.

(D) Encapsulation efficiency of DFI-containing SM-102 LNPs. Data represent three measurements from two independent batches (mean ± s.e.m.). *P* values were calculated using a two-tailed unpaired *t*-test.

(E) LNP diameter (µm) and polydispersity index (PDI, %) measured by dynamic light scattering. Grey lines represent individual batches; the batch mean is shown in green.

(F) IVIS luciferase imaging of mice 8 h after intravenous administration of NaCl or 5 µg Luc mRNA-LNPs (n = 4).

(G) IVIS luciferase imaging of explanted organs 8 h after intravenous administration of NaCl or 5 µg Luc

mRNA-LNPs (n = 2).

(H) Multiplex cytokine profiling of peripheral blood from aged (72-week-old) mice treated for 28 days with NaCl (n = 10), DFI mRNA-LNPs (n = 9), or recombinant IL-7/FLT3-L protein (n = 10). Average concentrations for each analyte are shown (raw data in Table S2).

(I) Z-score-scaled cytokine concentrations in peripheral blood from aged (72-week-old) mice treated for 28 days with NaCl (n = 5), Luc (n = 5), IL-7 (n = 5), Dll1 (n = 5), Flt3l (n = 5), or DFI (n = 4) mRNA-LNPs. Raw data are provided in Table S3.

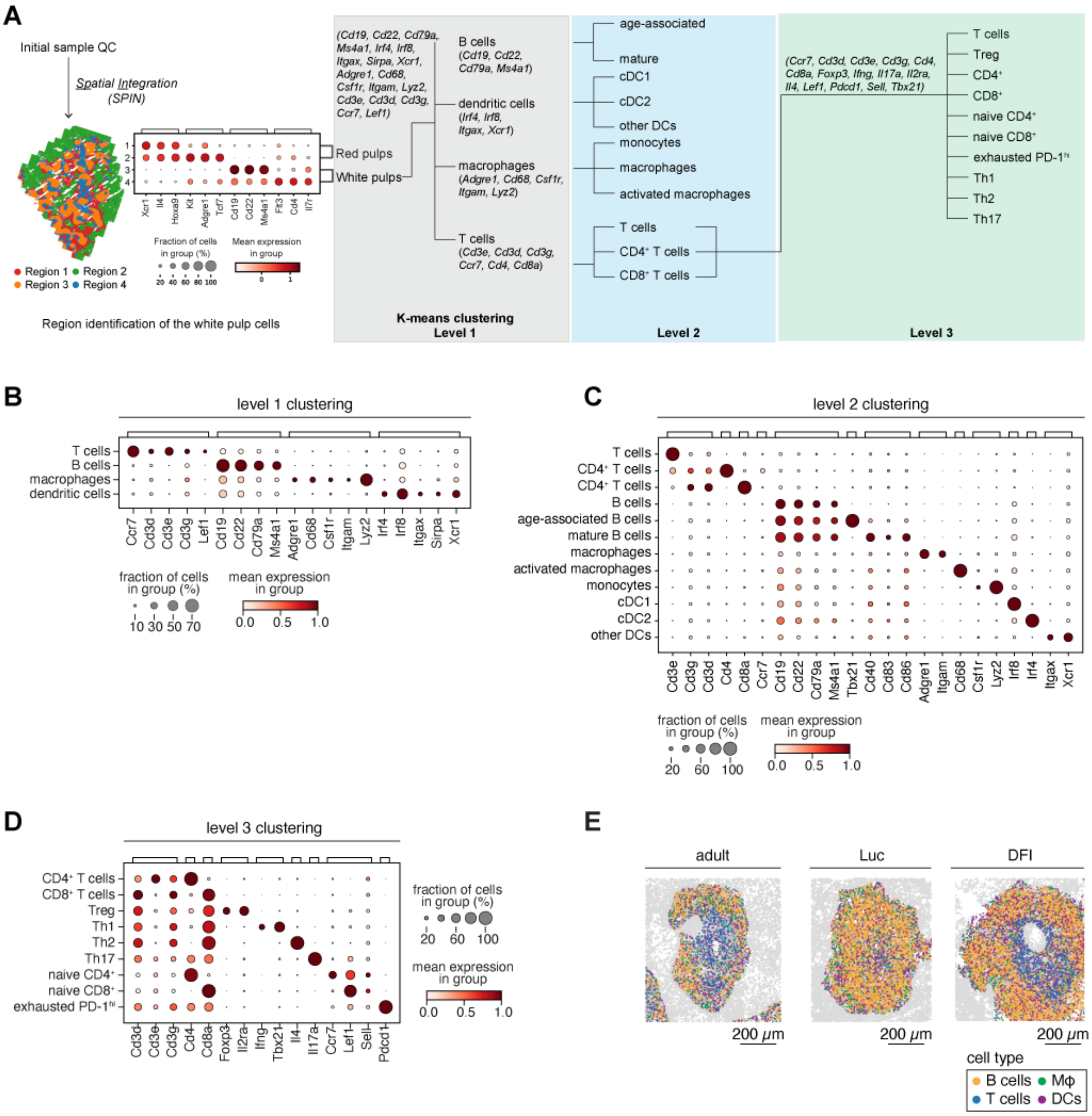

Supplementary Fig. 2 | STARmap PLUS profiling of spleen composition and transcriptional states following DFI treatment.

(A) Computational workflow for STARmap-based splenocyte cell typing. Cells were spatially clustered by the SPIN (spatial integration) toolkit; cells in white pulp regions were further subdivided by *k*-means using indicated marker genes.

(B-D) Gene expression dot plots from level 1, 2, and 3 clustering of major splenic cell types.

(E) Representative 2D projections showing spatial distribution of annotated cell types within white pulps of adult, Luc-treated, and DFI-treated aged mice.

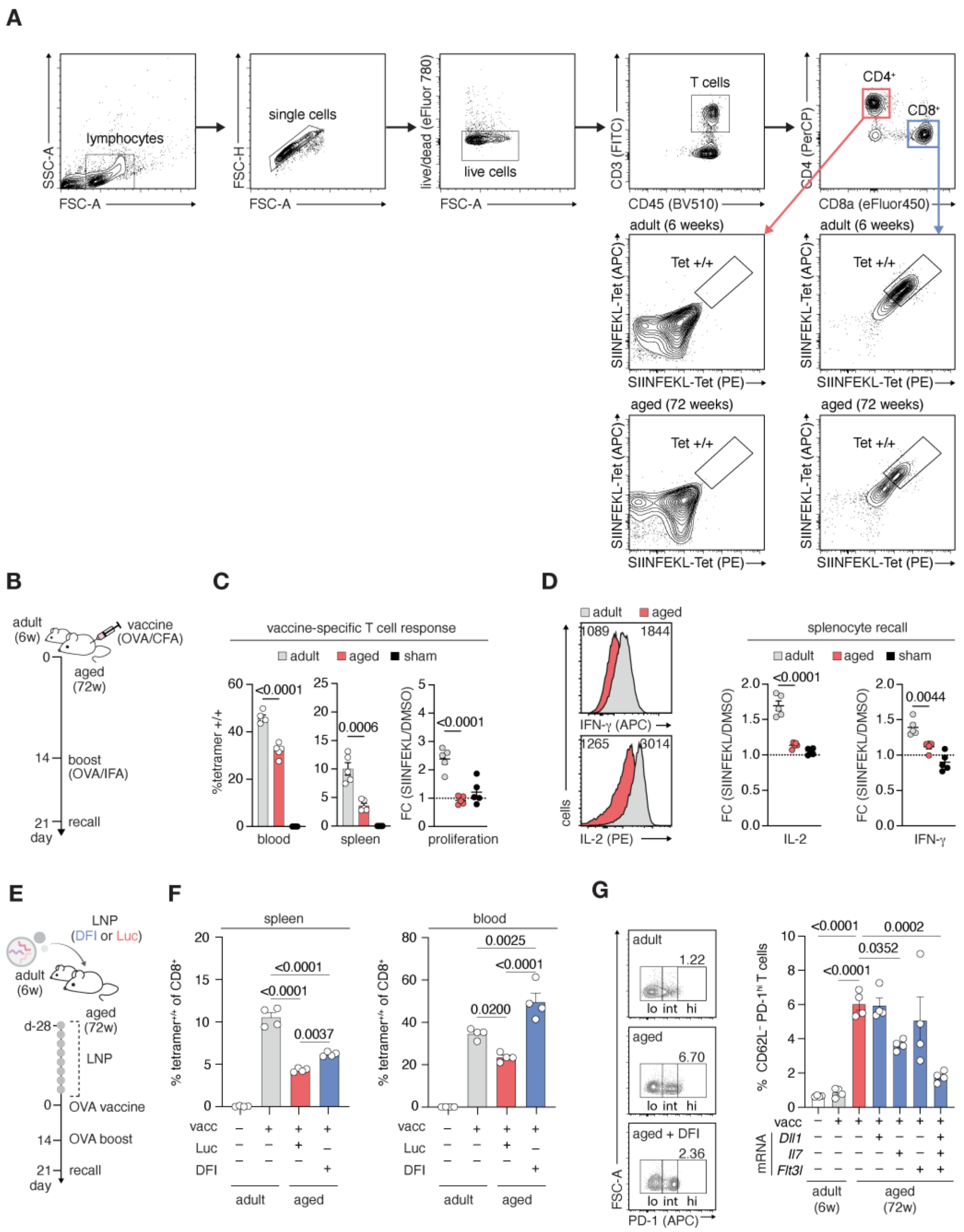

Supplementary Fig. 3 | Age-associated impairment of vaccine-induced CD8⁺ T cell responses and restoration by DFI.

(A) Representative flow cytometry gating strategy to identify SIINFEKL-H-2K^b tetramer-binding CD8⁺ T cells in spleen and blood from adult (6-week-old) and aged (72-week-old) mice.

(B) Experimental timeline for subcutaneous (s.c.) ovalbumin (OVA) peptide vaccination in adult and aged C57BL/6J mice, followed by *ex vivo* splenocyte recall with SIINFEKL peptide and intracellular cytokine staining (ICS).

(C) Frequencies of SIINFEKL-H-2K^b tetramer-binding T cells in peripheral blood (left) and spleen (middle). Fold change in total T cell counts after 24 h ex vivo SIINFEKL recall relative to vehicle (right).

Each dot represents one mouse (n = 5 per group). Data are mean ± s.e.m. *P* values were determined by one-way ANOVA with Tukey's post hoc test.
(D) Fold change in IL-2 and IFN-γ expression (mean fluorescence intensity, MFI) in splenocytes following 6 h ex vivo SIINFEKL recall relative to vehicle. n = 5 per group. Data are mean ± s.e.m. *P* values from one-way ANOVA with Tukey's post hoc test.
(E) Experimental workflow for 28-day conditioning with DFI or Luc mRNA-LNPs prior to adjuvanted OVA vaccination.
(F) Frequencies of SIINFEKL-H-$2K^b$ tetramer-binding T cells in spleen (left) and blood (right) 7 days post-vaccination (n = 4 per group).
(G) Frequencies of exhausted ($CD62L^-$ PD-1^{hi}) $CD8^+$ T cells in spleens (n = 4 per group). Data in **(F-G)** are mean ± s.e.m. *P* values were calculated by one-way ANOVA with Tukey's post hoc test.

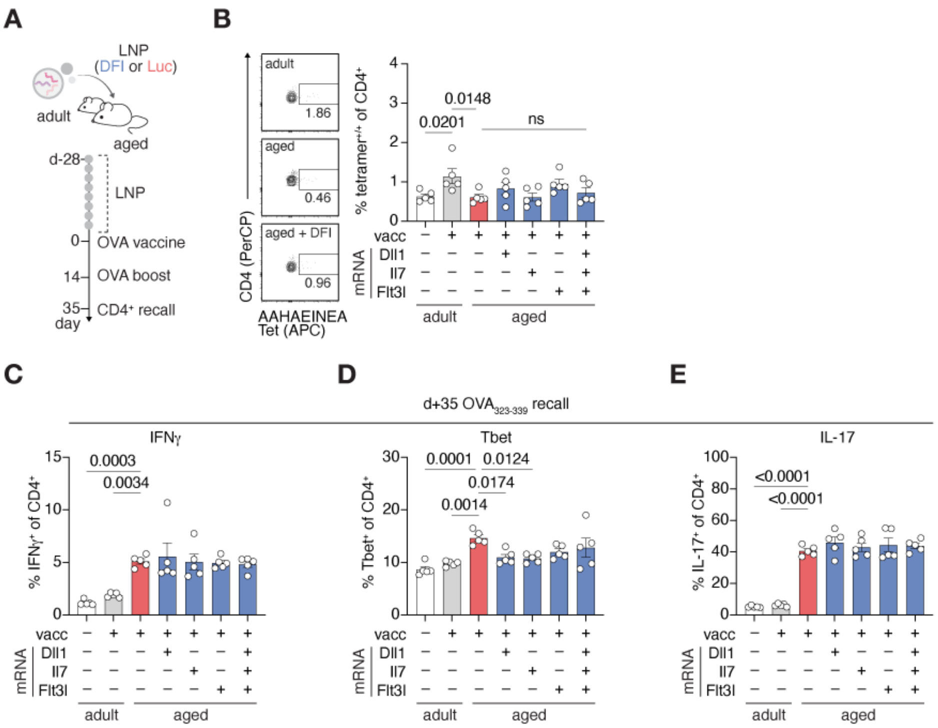

Supplementary Fig. 4 | DFI conditioning affects humoral, but not T helper responses to vaccination.
(A) Experimental workflow for 28-day DFI or control mRNA-LNP treatment followed by s.c. immunization with adjuvanted OVA protein. Immune responses were analyzed 35 days post-initial immunization.
(B) Frequencies of I-A^{b} tetramer-binding CD4^{+} T cells specific for the immunodominant OVA peptide OVA$_{323\text{-}339}$ (AAHAEINEA) (n = 5 per group).
(C-E) Frequencies of IFN-γ^{+}, T-bet^{+}, and IL-17^{+} CD4^{+} T cells following *ex vivo* OVA recall stimulation (n = 5 per group).

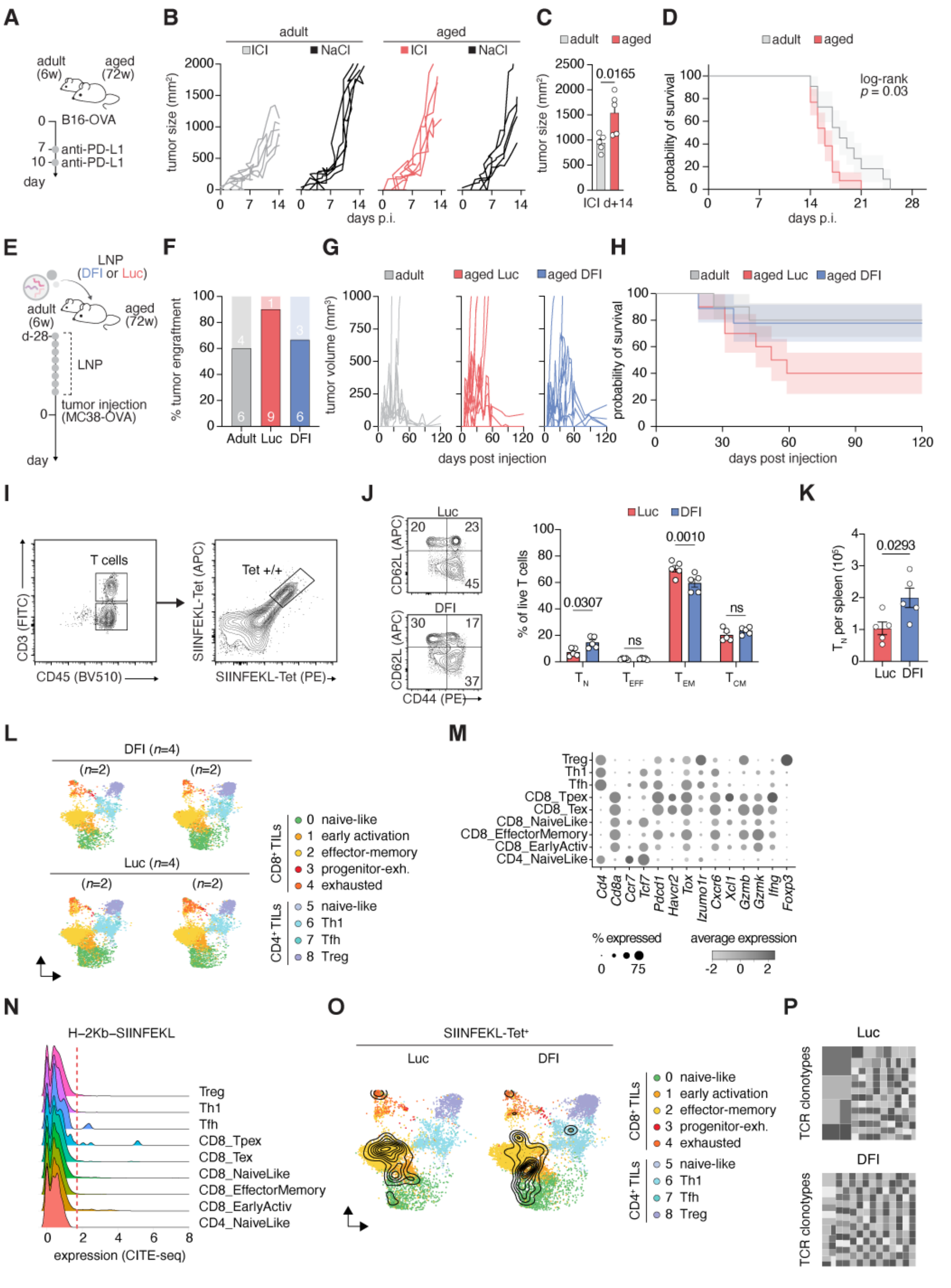

Supplementary Fig. 5 | DFI enhances age-impaired anti-tumor immunity and reshapes intratumoral T cell responses.

(A) Experimental design: adult (6-week-old) and aged (72-week-old) mice were subcutaneously

implanted with B16-OVA melanoma and treated with anti-PD-L1 antibody or vehicle on days 7 and 10 post-implantation.
(B-C) Longitudinal tumor growth curves (**B**) and tumor volumes at day 14 (**C**). Each line or dot represents one mouse.
(D) Kaplan-Meier survival curves (n = 5 per group). *P* value from log-rank (Mantel-Cox) test.
(**E**) Experimental design: aged mice received DFI or control (Luc) LNPs for 28 days, followed by subcutaneous MC38-OVA tumor challenge.
(**F**) Tumor engraftment rates across groups (n=10 adult, 10 aged + Luc, 9 aged + DFI).
(**G**) Tumor growth curves showing individual trajectories; same n as (B).
(**H**) Kaplan–Meier survival analysis for MC38-OVA model; n.s. p values by Log-rank test.
(I) Representative gating strategy for SIINFEKL-H-2K^b tetramer staining of CD8$^+$ tumor-infiltrating lymphocytes (TILs).
(J-K) Frequencies (**J**) and absolute counts (**K**) of naive (CD44$^-$ CD62L$^+$) splenic CD8$^+$ T cells in tumor-bearing mice (n = 5 per group).
(L-M) UMAP projections (**L**) and marker gene expression (**M**) from single-cell RNA-seq of CD45$^+$CD3$^+$ TILs (n = 34,104 cells from n = 8 mice).
(N-O) Histogram of H-2K^b-SIINFEKL tetramer oligo counts (**N**) and density maps of SIINFEKL-specific CD8$^+$ TCR clones overlaid on UMAP projections (**O**).
(P) Treemaps depicting frequencies of SIINFEKL-specific CD8$^+$ TCR clones in Luc- and DFI-treated mice.

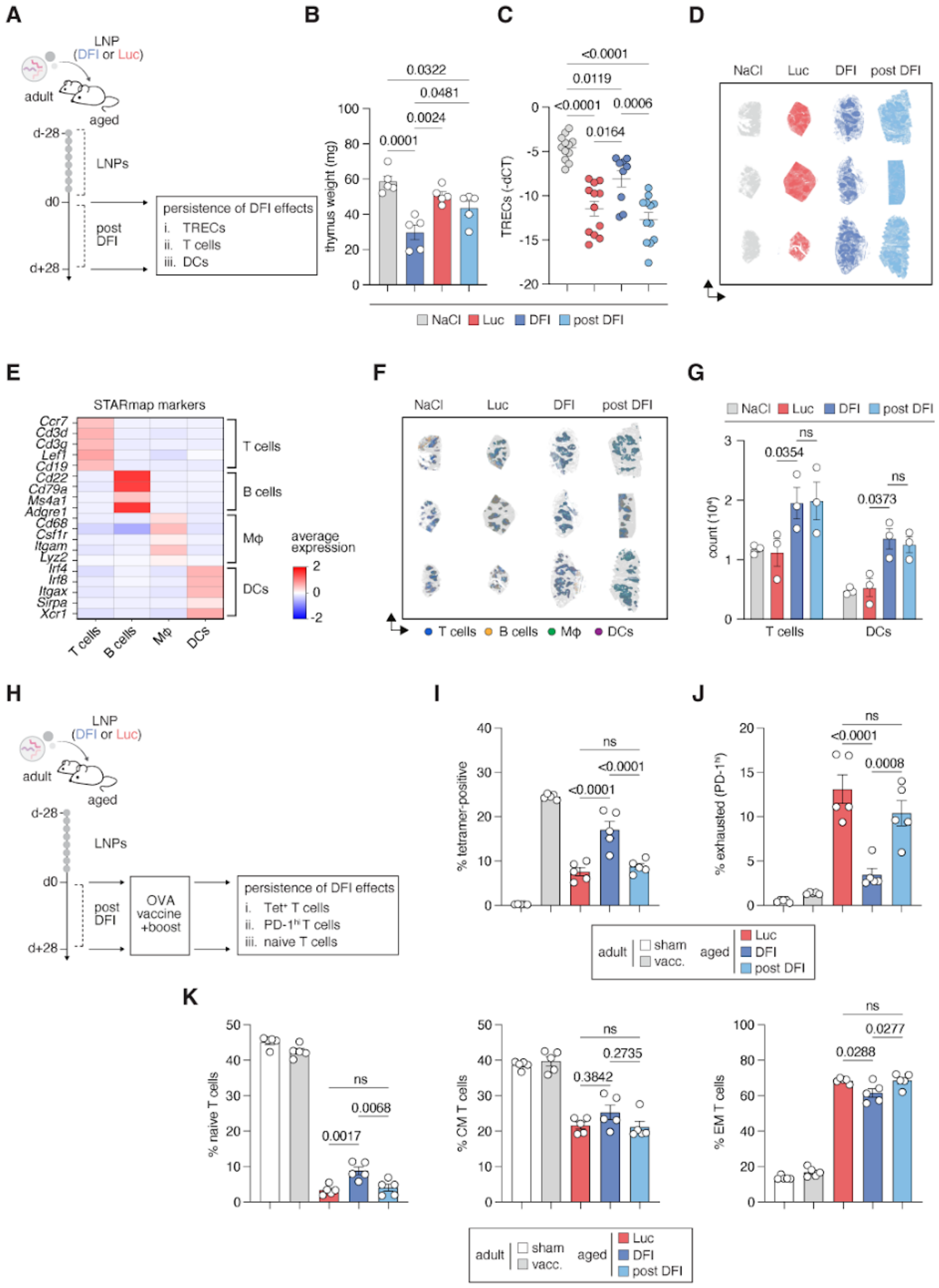

Supplementary Fig. 6 | Persistence and reversibility of immune effects following DFI withdrawal.
(A) Experimental workflow: 28 days of DFI or Luc control mRNA-LNP treatment followed by a 28-day washout. Assessments were performed on day 28 of treatment and after washout.
(B) Thymus weights at day 28 (n = 5 per group).
(C) Signal joint T cell receptor excision circle (sjTREC) quantification in peripheral blood (n = 12 adult,

12 aged + Luc, 9 aged + DFI, 12 aged post-DFI).
(D-F) STARmap analysis of splenic architecture: **(D)** 2D cell type projections, **(E)** marker gene expression dot plots, and **(F)** spatial distribution of annotated immune subsets (n = 3 per group).
(G) Absolute splenic counts of total T cells and dendritic cells (DCs) (n = 3 per group).
(H) Experimental workflow for post-DFI OVA protein vaccination.
(I-K) Frequencies of SIINFEKL-H-2K^b tetramer-binding CD8$^+$ T cells (**I**), exhausted CD62L$^-$ PD-1^{hi} CD8$^+$ T cells (**J**), and naive, central memory, and effector memory CD8$^+$ subsets (**K**) (n = 5 per group). Data throughout are mean ± s.e.m. *P* values were calculated using one-way ANOVA with Tukey's post hoc test.

Supplementary Data Tables

Table S1. RIBOmap targets and probe sequences.

Table S2. Raw values of serum cytokine measurements performed from peripheral blood of DFI mRNA-LNP, recombinant protein, or NaCl-treated animals.

Table S3. Raw values of serum cytokine measurements performed from peripheral blood of DFI mRNA-LNP, Luc mRNA-LNP, single DFI factor mRNA LNP, or NaCl-treated animals.

Table S4. STARmap targets and probe sequences.
