# Supplementary material for: Transient hepatic reconstitution of trophic factors enhances aged immunity
Source: Nature. 2025 Dec 17;650(8101):481–9. doi: 10.1038/s41586-025-09873-4 (PMC12893904; doi:10.1038/s41586-025-09873-4)
Supplement: Supplementary file 2 — Reporting Summary [file 41586_2025_9873_MOESM2_ESM.pdf]

nature portfolio

Corresponding author(s): Feng Zhang, PhD

Last updated by author(s): Oct 26, 2025

Reporting Summary

Nature Portfolio wishes to improve the reproducibility of the work that we publish. This form provides structure for consistency and transparency in reporting. For further information on Nature Portfolio policies, see our Editorial Policies and the Editorial Policy Checklist.

Statistics

For all statistical analyses, confirm that the following items are present in the figure legend, table legend, main text, or Methods section.

n/a	Confirmed	
☐	☒	The exact sample size (n) for each experimental group/condition, given as a discrete number and unit of measurement
☐	☒	A statement on whether measurements were taken from distinct samples or whether the same sample was measured repeatedly
☐	☒	The statistical test(s) used AND whether they are one- or two-sided<br>*Only common tests should be described solely by name; describe more complex techniques in the Methods section.*
☐	☒	A description of all covariates tested
☐	☒	A description of any assumptions or corrections, such as tests of normality and adjustment for multiple comparisons
☐	☒	A full description of the statistical parameters including central tendency (e.g. means) or other basic estimates (e.g. regression coefficient) AND variation (e.g. standard deviation) or associated estimates of uncertainty (e.g. confidence intervals)
☐	☒	For null hypothesis testing, the test statistic (e.g. F, t, r) with confidence intervals, effect sizes, degrees of freedom and P value noted<br>*Give P values as exact values whenever suitable.*
☒	☐	For Bayesian analysis, information on the choice of priors and Markov chain Monte Carlo settings
☒	☐	For hierarchical and complex designs, identification of the appropriate level for tests and full reporting of outcomes
☐	☒	Estimates of effect sizes (e.g. Cohen's d, Pearson's r), indicating how they were calculated

Our web collection on statistics for biologists contains articles on many of the points above.

Software and code

Policy information about availability of computer code

Data collection	No custom software was used for data collection.

Data analysis	Raw sequencing data were processed and aligned to the mause genome (GRCm39 - mm39) using the CellRanger pipeline (10x Genomics, version 7.1.0).<br><br>For IVIS imaging, the Aura imaging software (v4.0) was used.<br><br>The mouse genome assembly (GRCm39) was used for data alignment (NCBI RefSeq assembly; GCF_000001635.27).<br><br>For spatial sequencing data, we used the Squidpy (v1.4.0) integration of CellPhoneDB and Omnipath to identify shifts in receptor-ligand interactions at each time point, which are each publicly available.<br><br>For single-cell sequencing data, we used Seurat v5.3.0 (for single-cell sequencing analysis), Harmony v1.0 (for single-cell data integration), Scanpy vl.8.2 and Matplotlib v3.8, which are each publicly available.<br><br>STARmap data analysis was performed similarly to Wang et al and Shi et al. Image deconvolution was achieved with Huygens Essential version 21.04 (Scientific Volume lmaging, The Netherlands, http://svi.nl), using the CMLE algorithm, with SNR:10 and 10 iterations. Image registration, spot calling, and barcode filtering were performed as previously described by the above-mentioned publications.<br><br>All code necessary to reproduce all spatial thymus RNA-seq analyses is available at: https://github.com/immunoliugy/aging_mouse_thymus.<br>All code necessary to reproduce all RIBOmap / STARmap PLUS analyses is available at: https://github.com/wanglab-broad/hepatic_recon.

For manuscripts utilizing custom algorithms or software that are central to the research but not yet described in published literature, software must be made available to editors and reviewers. We strongly encourage code deposition in a community repository (e.g. GitHub). See the Nature Portfolio guidelines for submitting code & software for further information.

Data

Policy information about availability of data

All manuscripts must include a data availability statement. This statement should provide the following information, where applicable:

- Accession codes, unique identifiers, or web links for publicly available datasets
- A description of any restrictions on data availability
- For clinical datasets or third party data, please ensure that the statement adheres to our policy

All single-cell profiling data of circulating T cells and B16-OVA TILs in aging mice are available at Zenodo (accession number 17385897). All spatial profiling (RIBOmap / STARmap PLUS) data of liver and spleen samples are available at Zenodo (accession number 13868686). All spatial profiling (Slide-TCR-seq) data of thymuses across age are available at Zenodo (accession number 17525728). The processed spatial thymus dataset across age is publicly available at the Broad Single Cell Portal (accession number SCP2424). The mouse genome assembly (GRCm39) was used for data alignment (NCBI RefSeq assembly; GCF_000001635.27).

Research involving human participants, their data, or biological material

Policy information about studies with human participants or human data. See also policy information about sex, gender (identity/presentation), and sexual orientation and race, ethnicity and racism.

Reporting on sex and gender	No human participants, their materials, or their data were included in this study.
Reporting on race, ethnicity, or other socially relevant groupings	No human participants, their materials, or their data were included in this study.
Population characteristics	No human participants, their materials, or their data were included in this study.
Recruitment	No human participants, their materials, or their data were included in this study.
Ethics oversight	No human participants, their materials, or their data were included in this study.

Note that full information on the approval of the study protocol must also be provided in the manuscript.

Field-specific reporting

Please select the one below that is the best fit for your research. If you are not sure, read the appropriate sections before making your selection.

☒ Life sciences ☐ Behavioural & social sciences ☐ Ecological, evolutionary & environmental sciences

For a reference copy of the document with all sections, see nature.com/documents/nr-reporting-summary-flat.pdf

Life sciences study design

All studies must disclose on these points even when the disclosure is negative.

Sample size	The sample size for vaccination experiments was decided based on prior publications with similar experiments [Zhivaki, D. et al. Correction of age-associated defects in dendritic cells enables CD4+ T cells to eradicate tumors. Cell 187, 3888–3903.e18 (2024)].

The sample size for tumor experiments was decided based on prior publications with similar experiments [Tsukamoto, H. et al. Aging-associated and CD4 T-cell-dependent ectopic CXCL13 activation predisposes to anti-PD-1 therapy-induced adverse events. Proc. Natl. Acad. Sci. U. S. A. 119, e2205378119 (2022) and Georgiev, P. et al. Age-associated contraction of tumor-specific T cells impairs antitumor immunity. Cancer Immunol. Res. 12, 1525–1541 (2024)].

The sample size for NOD experiments was decided based on prior publications with similar experiments [Trudeau, J. D. et al. Prediction of spontaneous autoimmune diabetes in NOD mice by quantification of autoreactive T cells in peripheral blood. J. Clin. Invest. 111, 217–223 (2003)].

The sample size for EAE experiments was decided based on prior publications with similar experiments [Sanmarco, L. M. et al. Lactate limits CNS autoimmunity by stabilizing HIF-1α in dendritic cells. Nature 620, 881–889 (2023)].

For all other exploratory experiments, no sample size calculations were performed.

Data exclusions

For animal experiments other than survival studies, mice diseased during the experiment were excluded from the analyses. From longitudinal single-cell RNA/V(D)J sequencing experiments, one sampling timepoint was excluded.

Replication

All attempts at replication were successful. Replicated experiments were identically or similarly designed. In vivo experiments were replicated using the same mouse strains and facilities at the Broad Institute. NOD and EAE experiments were not repeated.

Randomization

For all experiments, allocation of mice into experimental groups was randomized after stratifying for age and sex.

Blinding

Separate investigators performed treatment and data collection. Data collecting investigators, e.g., for tumor size measurements, were blinded to the treatment groups. Data analyzing investigators were not blinded to the treatment groups, as they involved internal controls, with the exception of pathologists for toxicity studies, who were blinded for the analyses. Experimental and control animals were treated equally and, when possible, housed in mixed cages.

Reporting for specific materials, systems and methods

We require information from authors about some types of materials, experimental systems and methods used in many studies. Here, indicate whether each material, system or method listed is relevant to your study. If you are not sure if a list item applies to your research, read the appropriate section before selecting a response.

Materials & experimental systems

n/a	Involved in the study	
☐	☒	Antibodies
☐	☒	Eukaryotic cell lines
☒	☐	Palaeontology and archaeology
☐	☒	Animals and other organisms
☒	☐	Clinical data
☒	☐	Dual use research of concern
☒	☐	Plants

Methods

n/a	Involved in the study	
☒	☐	ChIP-seq
☐	☒	Flow cytometry
☒	☐	MRI-based neuroimaging

Antibodies

Antibodies used

Immunohistochemistry, immunofluorescence and STARmap analyses of DLL1: anti-DLL1 antibody 1:800 (Abcam ab10554) and AF546-labeled secondary donkey anti-rabbit IgG antibody 1:500 (Invitrogen A10040).

Mouse T cell analyses: anti-mouse IL-2 PE 1:100 (Biolegend; 503808; clone JES6-5H4; lot B351622, B377599), anti-mouse CD45 BV510 1:100 (Biolegend; 103138; clone 30-F11; lot B386738, B360620, B384034), anti-mouse IFN-γ APC 1:100 (Biolegend; 505810; clone XMG1.2; lot B354911, B370994, B396246), anti-mouse/human CD44 PE 1:100 (Biolegend; 103024; clone IM7; lot B343363), Anti-Mo CD8a eBioscience eFluor 450 1:100 (Invitrogen; 48-0081-82; clone 53-6.7; lot 2527379), Rat Anti-Mouse CD4 PerCP 1:100 (BD Bioscience; 553052; clone RM4-5; lot 2279727, 3201956, 1334056), anti-mouse CD62L APC 1:100 (Biolegend; 104412; clone MEL-14; lot B371017), anti-mouse CD3 FITC 1:100 (Biolegend; 100204; clone 17A2; lot B388315, B388061, B406287), Hamster Anti-Mouse TCRβ APC 1:100 (BD bioscience; 553174; clone H57-597; lot 3030398, 2076848), TruStain FcX anti-mouse CD16/32 1:50 (Biolegend; 101320; clone 93; lot B398113, B380119, B368516, B372578, B419152), eBioscience Fixable Viability Dye eFluor 780 1:1000 (Invitrogen; 65-0865-14; lot 2752774), anti-mouse CD4 PE-Cy5.5 1:500 (Biolegend; 100514; clone RM4-5; lot B398342), anti-mouse CD8a BV510 1:100 (Biolegend; 100752; clone 53-6.7; lot B427044), anti-mouse/human CD44 PE 1:200 (Biolegend; 103024; clone IM7; lot B343363), anti-mouse CD62L APC 1:100 (Biolegend; 104412; clone MEL-14; lot B371017), anti-mouse CD3 Pacific Blue 1:100 (Biolegend; 100214; clone 17A2; lot B427533), anti-mouse CD279 (PD-1) APC 1:100 (Biolegend; 135210; clone 29F.1A12; lot B376789).

Mouse thymus analyses: anti-mouse CD4 PE-Cy5.5 1:500 (Biolegend; 100514; clone RM4-5; lot B398342), anti-mouse CD8a BV510 1:100 (Biolegend; 100752; clone 53-6.7; lot B427044), anti-mouse CD25 PerCP 1:200 (Biolegend; 102028; clone PC61; lot B378943), anti-mouse/human CD44 PE 1:200 (Biolegend; 103007; clone IM7; lot B417002), Rat Anti-Mouse CD117 APC 1:100 (BD bioscience; 553356; clone 2B8; lot 3199305), anti-mouse CD28 FITC 1:100 (Biolegend; 122008; clone E18; lot B373960), anti-mouse CD24 Pacific Blue 1:500 (Biolegend; 101820; clone M1/69; lot B385383), anti-mouse TCR β chain PE/Cyanine7 1:400 (Biolegend; 109222; clone

H57-597; lot B394527), Rat Anti-Mouse CD45R/B220 BUV661 1:100 (Bd bioscience; 612972; clone RA3-6B2; lot 3292032), TruStain FcX anti-mouse CD16/32 1:50 (Biolegend; 101320; clone 93; lot B398113, B380119, B368516, B372578, B419152), eBioscience Fixable Viability Dye eFluor 780 1:1000 (Invitrogen; 65-0865-14; lot 2752774).

Tetramer stainings:
For OVA vaccination experiments, SIINFEKL-H-2K^b-PE and SIINFEKL-H-2K^b-APC tetramers were used at 1:100 dilution and AAHAEINEA-I-A^b-PE and AAHAEINEA-I-A^b-APC tetramers were used at 1:20 dilution. For central tolerance experiments in Act-mOVA mice, the same SIINFEKL-H-2K^b tetramers (1:100 dilution) were used in combination with AAHAEINEA-I-A^b-PE and AAHAEINEA-I-A^b-APC tetramers (1:20 dilution). Non vaccinated wild-type T cells (negative) and OT-I and OT-II T cells (positive) were included as staining controls for MHC class I and II tetramers, respectively.

For autoimmunity experiments in NOD mice, KYNKANAFL-H-2K^d-PE and KYNKANAFL-H-2K^d-APC tetramers were used at 1:50 dilution, with NY8.3 T cells serving as positive controls for staining.

For EAE experiments in C57BL/6J mice, GWYRSPFSRVVH-I-A^b-PE and GWYRSPFSRVVH-I-A^b-APC tetramers were used at 1:25 dilution. Control tetramers consisted of I-A^b-restricted human CLIP87-101 (PVSKMRMATPLLMQA) conjugated to PE and APC, also at 1:25 dilution.

Validation

All antibodies and tetramers used in this study have been previously validated by commercial manufactures, previous publications, and/or this study.

Immunohistochemistry, immunofluorescence and STARmap analyses of DLL1:
anti-DLL1 antibody (Abcam ab10554), reported to recognize mouse, human and rat DLL1 (https://www.abcam.com/en-us/products/primary-antibodies/dll1-antibody-ab10554)
AF546-labeled secondary donkey anti-rabbit IgG antibody, reported to recognized rabbit IgG (manufacturer's website)

Flow cytometry and fluorescence-activated cell sorting:
PE anti-mouse IL-2 Antibody (Biolegend; 503808), reported to recognize mouse IL-2 (https://www.biolegend.com/en-us/products/pe-anti-mouse-il-2-antibody-954)
Violet 510™ anti-mouse CD45 Antibody BV510 (Biolegend; 103138), reported to recognize mouse CD45 (https://www.biolegend.com/en-us/products/brilliant-violet-510-anti-mouse-cd45-antibody-7995)
APC anti-mouse IFN-γ Antibody (Biolegend; 505810), reported to recognize mouse IFN- γ (https://www.biolegend.com/en-us/products/apc-anti-mouse-ifn-gamma-antibody-993)
PE anti-mouse/human CD44 Antibody (Biolegend; 103024), reported to recognize mouse/human CD44 (https://www.biolegend.com/en-us/products/pe-anti-mouse-human-cd44-antibody-2206)
CD8a Monoclonal Antibody (53-6.7), eFluor 450 (Invitrogen; 48-0081-82), reported to recognize mouse CD8a (https://www.thermofisher.com/antibody/product/CD8a-Antibody-clone-53-6-7-Monoclonal/48-0081-82)
Phamingen PerCP Rat Anti-Mouse CD4 (BD Bioscience; 553052), reported to recognize mouse (https://www.bdbiosciences.com/en-de/products/reagents/flow-cytometry-reagents/research-reagents/single-color-antibodies-ruo/percp-rat-anti-mouse-cd4.553052?tab=product_details)
FITC anti-mouse CD3 Antibody (Biolegend; 100204), reported to recognize mouse CD3 (https://www.biolegend.com/nl-be/products/fitc-anti-mouse-cd3-antibody-45)
BD Pharmingen APC Hamster Anti-Mouse TCR ß Chain (BD bioscience; 553174), reported to recognize mouse TCRβ (https://www.bdbiosciences.com/en-de/products/reagents/flow-cytometry-reagents/research-reagents/single-color-antibodies-ruo/apc-hamster-anti-mouse-tcr-chain.553174?tab=product_details)
APC anti-mouse CD62L Antibody (Biolegend; 104412), reported to recognize mouse CD62L (https://www.biolegend.com/en-us/products/apc-anti-mouse-cd62l-antibody-381)
Pacific Blue anti-mouse CD3 (Biolegend; 100214), reported to recognize mouse CD3 (https://www.biolegend.com/en-us/products/pacific-blue-anti-mouse-cd3-antibody-3317)
APC anti-mouse CD279 /PD-1) Antibody (PD-1) APC (Biolegend; 135210), reported to recognize mouse CD279 (https://www.biolegend.com/en-us/products/apc-anti-mouse-cd279-pd-1-antibody-6497)
PE/Cyanine5 anti-mouse CD4 Antibody (Biolegend; 100514), reported to recognize mouse CD4 (https://www.biolegend.com/en-us/products/pe-cyanine5-anti-mouse-cd4-antibody-483)
Violet 510 anti-mouse CD8a Antibody (Biolegend; 100752), reported to recognize mouse CD8a (https://www.biolegend.com/en-us/products/brilliant-violet-510-anti-mouse-cd8a-antibody-7992)
PerCP anti-mouse CD25 Antibody (Biolegend; 102028), reported to recognize mouse CD25 (https://www.biolegend.com/en-us/products/percp-anti-mouse-cd25-antibody-4263)
PE anti-mouse/human CD44 Antibody (Biolegend; 103007), reported to recognize mouse/human CD44 (https://www.biolegend.com/en-us/products/pe-anti-mouse-human-cd44-antibody-2206)
Rat Anti-Mouse CD117 APC (BD bioscience; 553356), reported to recognize mouse CD117
FITC anti-mouse CD28 Antibody (Biolegend; 122008), reported to recognize mouse CD28 (https://www.biolegend.com/en-us/products/fitc-anti-mouse-cd28-antibody-3777)
Pacific Blue anti-mouse CD24 Antibody (Biolegend; 101820), reported to recognize mouse CD24 (https://www.biolegend.com/en-us/products/pacific-blue-anti-mouse-cd24-antibody-3584)
PE/Cyanine7 anti-mouse TCR ß chain Antibody (Biolegend; 109222), reported to recognize mouse TCR β chain (https://www.biolegend.com/en-us/products/pe-cyanine7-anti-mouse-tcr-beta-chain-antibody-4144)
Horizon BUV661 Rat Anti-Mouse CD45/RB220 (BD Bioscience; 612972), reported to recognize mouse CD45R/B220 BUV661 (https://www.bdbiosciences.com/en-de/products/reagents/flow-cytometry-reagents/research-reagents/single-color-antibodies-ruo/buv661-rat-anti-mouse-cd45r-b220.612972?tab=product_details)
(c-Kit) Monoclonal Antibody (2B8), APC-eFluor 780 (ThermoFischer; 47-1171-82), reported to recognize mouse and pig cKit (https://www.thermofisher.com/antibody/product/CD117-c-Kit-Antibody-clone-2B8-Monoclonal/47-1171-82)
BD Horizon BUV395 Rat Anti-Mouse Ly-6A/E (BD; 744328), reported to recognize mouse SCA1 (https://www.bdbiosciences.com/en-de/products/reagents/flow-cytometry-reagents/research-reagents/single-color-antibodies-ruo/buv395-rat-anti-mouse-ly-6a-e.563990?tab=product_details)
BD Horizon BUV737 Mouse Anti-Human CD45 (367-0451-82), reported to recognize mouse CD45 (https://www.bdbiosciences.com/

en-de/products/reagents/flow-cytometry-reagents/research-reagents/single-color-antibodies-ruo/buv737-mouse-anti-human-cd45.568524?tab=product_details)
BD OptiBuild BV650 Rat Anti-Mouse CD41 (BD; 740504), reported to recognize mouse CD41 (https://www.bdbiosciences.com/en-eu/products/reagents/flow-cytometry-reagents/research-reagents/single-color-antibodies-ruo/BV650-Rat-Anti-Mouse-CD41.740504?tab=product_details)
APC anti-mouse CD127 (IL-7Ra) Antibody (BioLegend; 135012), reported to recognize mouse IL7Ra (https://www.biolegend.com/en-us/products/apc-anti-mouse-cd127-il-7ralpha-antibody-6191)
anti-mouse FLT3 PerCP-eFluor710 (eBioscience; 46-1351-82), reported to recognize mouse FLT3 (https://www.thermofisher.com/antibody/product/CD135-Flt3-Antibody-clone-A2F10-Monoclonal/46-1351-82)
PE/Cyanine7 anti-mouse CD150 (SLAM) Antibody BioLegend; 115914), reported to recognize mouse CD150 (https://www.biolegend.com/en-us/products/pe-cyanine7-anti-mouse-cd150-slam-antibody-3056)
TruStain FcX (anti-mouse CD16/32) Antibody (Biolegend; 101320), reported to recognize mouse CD16/32 (https://www.biolegend.com/en-us/products/trustain-fcx-anti-mouse-cd16-32-antibody-5683)
Invitrogen eBioscience Fixable Viability Dye eFluor 780 (Invitrogen; 65-0865-14), reported to recognize live cells from dead cells (https://www.fishersci.de/shop/products/fixable-viability-dye-efluor-780-1/13539140?srsltid=AfmBOopvFHFehhCHI9VFYBg8JDn94rU93NlIL6IKEPFjlz-I1kTm4P18)

SIINFEKL-H-2 Kb – PE, validated internally by isolating T cells from OT-I mouse as staining control
SIINFEKL-H-2 Kb – APC, validated internally by isolating T cells from OT-I mouse as staining control
AAHAEINEA-I-A b – PE, validated internally by isolating T cells from OT-II mouse as staining control
AAHAEINEA-I-A b – PE, validated internally by isolating T cells from OT-II mouse as staining control
KYNKANAFL-H-2 Kd – PE, validated internally by isolating T cells from NY8.3 mouse as staining control
KYNKANAFL-H-2 Kd – APC, validated internally by isolating T cells from NY8.3 mouse as staining control

Eukaryotic cell lines

Policy information about cell lines and Sex and Gender in Research

Cell line source(s)	OVA-expressing melanoma B16 (B16-OVA) and MC38 (MC38-OVA) cell lines were kindly provided by Michael Kilian (Harvard Medical School, Boston, USA).
Authentication	None of the cell lines used were authenticated by our laboratory.
Mycoplasma contamination	The cell lines were not tested for mycoplasma contamination.
Commonly misidentified lines (See ICLAC register)	Not applicable.

Animals and other research organisms

Policy information about studies involving animals; ARRIVE guidelines recommended for reporting animal research, and Sex and Gender in Research

Laboratory animals

Wild-type C57BL/6J mice
For experiments in Figures 1-5 and associated extended data and supplementary figures: female and male C57BL/6J mice (Jackson Labs, strain #000664).
For experiments in Figures 1B-D and associated extended data and supplementary figures: age between 6 and 100 weeks (longitudinal experiment).
For experiments in Figure 2A and associated extended data and supplementary figures: age between 6 and 90 weeks (longitudinal experiment).
For experiments in Figures 2B,C,D,E,G,H,I,J,K,L and associated extended data and supplementary figures: age 6 weeks (adult) or 72 weeks (aged).
For experiments in Figures 3B,C,D,E;F,G,H and associated extended data and supplementary figures: age 6 weeks (adult) or 72 weeks (aged).
For experiments in Figure 3I and associated extended data and supplementary figures: age between 6 and 90 weeks (longitudinal experiment).
For experiments in Figures 4B,C,D,F,G,H,J,K,L,M,N,O,P,Q,R and associated extended data and supplementary figures: age 72 weeks (aged).
For all other experiments: age 6-12 weeks.

Transgenic mice
For experiments in Figure 2 and associated extended data figures: female and male C57BL/6-Tg(Nr4a1-EGFP/cre)820Khog/J (Nur77-GFP) mice (Jackson Labs, strain #016617; 6-12 weeks of age), FVB-Tg(Rag2-EGFP)1Mnz/J (Rag2-EGFP) mice (Jackson Labs, strain #005688; 6-12 weeks of age).
For experiments in Figure 5 and associated extended data figures: female NOD mice (Jackson Labs, strain #000664; 6 weeks of age at experiment start); female and male C57BL/6-Tg(CAG-OVAL)916Jen/J (Act-mOVA) mice (Jackson Labs, strain #005145; 6-12 weeks of age), female and male C57BL/6-Tg(TcraTcrb)1100Mjb/J (OT-I) mice (Jackson Labs, strain #003831; 6-12 weeks of age) female and male B6.Cg-Tg(TcraTcrb)425Cbn/J (OT-II) mice (Jackson Labs, strain #004194; 6-12 weeks of age).

Animals were kept on a 12-h light/dark cycle between 68 °F and 79 °F and 30–70% humidity. Mice were acclimated at the animal

	facility for at least 7 days before performing any experiments.
Wild animals	No wild animals were used in this study.
Reporting on sex	Except where noted and experimentally warranted (NOD and EAE experiments), all experiments were conducted with sex-matched animals, without bias to either sex. Sex-based analysis was not performed.
Field-collected samples	No field-collected samples were used in this study.
Ethics oversight	All animal experiments were approved by the Institutional Animal Care and Use Committee (IACUC) of the Broad Institute (Protocol ID 0017-09-14-2). Animal maintenance complied with all relevant ethical regulations and were consistent with local, state and federal regulations as applicable, including the National Institutes of Health Guide for the Care and Use of Laboratory Animals.

Note that full information on the approval of the study protocol must also be provided in the manuscript.

Plants

Seed stocks	No plants were used in this study.
Novel plant genotypes	No plants were used in this study.
Authentication	No plants were used in this study.

Flow Cytometry

Plots

Confirm that:

☒ The axis labels state the marker and fluorochrome used (e.g. CD4-FITC).

☒ The axis scales are clearly visible. Include numbers along axes only for bottom left plot of group (a 'group' is an analysis of identical markers).

☒ All plots are contour plots with outliers or pseudocolor plots.

☒ A numerical value for number of cells or percentage (with statistics) is provided.

Methodology

Sample preparation	Cells were prepared and stained according to the staining protocol of each experiment outlined in the methods section, pelleted at 500 g for 5min, and resuspended in 200 µL of flow cytometry buffer (PBS supplemented with 2% EDTA (Life Technologies 15575020) and 5% FBS (VWR 97068-085)). All antibodies and tetramers used can be found in the Reporting Summary.
Instrument	Samples were run for flow cytometry analysis on a Beckman Coulter Cytoflex LX flow cytometer. For FACS, cells were run either on a
Software	Analysis was performed using the FlowJo v10 software.
Cell population abundance	Cell population abundances are provided in the figures depicting gating strategies. For single-cell RNA-seq of purified T cells, post-sort purity was approximately 90%.
Gating strategy	FACS gating strategies for all analyses are supplied in Extended Data Figures and Supplementary Information. Population definitions are stated in the manuscript main text or methods. For analysis of non-lineage antibodies, the gating was determined by florescence-minus-one (FMO). For antigen-specific cell analysis, cells from naive mice, unvaccinated mice, Act-mOVA mice, OT-I or OT-II mice, were used as negative and positive controls for gating strategy, respectively.

☒ Tick this box to confirm that a figure exemplifying the gating strategy is provided in the Supplementary Information.
